# A Medicoeconomic Evaluation of a Telehealth Platform for Elective Outpatient Surgeries: Protocol for a Randomized Controlled Trial

**DOI:** 10.2196/44006

**Published:** 2023-04-24

**Authors:** Florian Robin, Maxim Roy, Alexandre Kuftedjian, Laurelie Perret, Frédéric Lavoie, Alexandre Castonguay, Marie-Pascale Pomey, Cedrick Zaouter, Guy Pare

**Affiliations:** 1 Department of Anesthesiology and Pain Medicine University of Montreal Montreal, QC Canada; 2 Department of Anesthesiology Centre hospitalier de l'Université de Montréal Montreal, QC Canada; 3 Research Center Centre hospitalier de l'Université de Montréal Montreal, QC Canada; 4 École des Hautes Études Commerciales Montréal, QC Canada; 5 School of Public Health University of Montreal Montréal, QC Canada

**Keywords:** outpatient surgery, cost-effectiveness, telehealth solution, surgery, technologies, quality, safety, support, patient, cost, telecare, telehealth, platform, development

## Abstract

**Background:**

The number of elective outpatient surgeries in Canada has increased markedly in the last 10 years. However, unanticipated cancellations on the day of surgery and adverse postoperative events are frequent. Modern technologies have been shown to be of great help in the medical field in improving patient care. Thus, it is likely that dedicated technologies could also significantly improve surgical outpatients’ pathways. Therefore, the department of anesthesiology at the University of Montreal Medical Center, in collaboration with LeoMed, a telemedicine platform, has developed a telehealth solution to offer more efficient perioperative support and follow-up for patients undergoing ambulatory surgery.

**Objective:**

The objective is to evaluate the medicoeconomic benefit of a dedicated perioperative telehealth platform for patients undergoing day surgery. Our hypothesis is that this dedicated telecare solution will allow more efficient patient care, which will reduce all types of medical costs related to day surgery pathways.

**Methods:**

This study is a single-center, single-blinded, 2-group randomized controlled trial. One thousand patients aged over 18 years with internet access who are scheduled to undergo ambulatory surgery will be enrolled and randomized either to follow a perioperative path that includes a patient-tailored perioperative digital app via the LeoMed telecare platform for 1 month or to follow the standard of care, which does not offer personalized digital support. The primary outcome will be to evaluate the cost-effectiveness of the telecare platform, assessing direct costs from factors such as unanticipated cancellations on the day of surgery due to preoperative instructions not being followed, calls to the local health information line, calls to the provincial health information line, emergency department consultations, unplanned readmissions, or medical visits for problems related to the surgical procedure within the first 30 days after the intervention. The secondary outcome will be to evaluate cost utility using a questionnaire assessing quality-adjusted life years. A blinded independent research team will analyze outcomes. All data will be analyzed according to the intention-to-treat principle. A sample size of 500 subjects in each group was calculated to detect a 21% reduction in postoperative complications with a power of 90%. This study has been approved by the ethics board of Centre hospitalier de l’Université de Montréal (University of Montreal Health Centre). No employee of LeoMed was involved in the study conception, and none will be involved in either data collection or analysis.

**Results:**

Results of this trial will be useful to determine the economic benefit of a telecare platform specifically developed for surgical outpatient pathways.

**Conclusions:**

We believe that the deployment of a dedicated perioperative telehealth app will lead to better patient care and fewer postoperative complications, which will lower all types of costs related to surgical outpatient care.

**Trial Registration:**

ClinicalTrials.gov NCT04948632; https://ClinicalTrials.gov/ct2/show/NCT04948632

**International Registered Report Identifier (IRRID):**

DERR1-10.2196/44006

## Introduction

Outpatient surgeries have increased significantly in the last 3 decades [1]. In Canada, there was an overall 30% increase in outpatient surgeries between the fiscal years 1995-1996 and 2005-2006 [2]. In some countries, day surgeries account for more than 70% of all elective procedures [3]. Nowadays, it is common to perform day surgeries for both higher-risk patients and procedures [4]. However, postoperative measures currently in place to support and follow patients undergoing day surgeries are suboptimal. Indeed, a recent Canadian study has shown that the same-day cancellation rate for outpatient surgeries ranges from 8% to 10% [5,6]. The authors of that study also mentioned that 60% of these cancellations could have been avoided with better preoperative support [7]. Despite current recommendations, adverse postoperative events are very rarely searched for or detected by health care institutions [8-10]. The most common reasons for readmission, emergency department consultation, or unplanned medical visits are postoperative pain, nausea, and vomiting [10]. In addition, the prevalence of moderate-to-severe postoperative pain after ambulatory surgery ranges from 21% to 30% at 24 hours, and as much as 24% to 43% at 7 days [11,12]. This could lead to inappropriate postoperative use of opioids [13]. Inadequate pain relief is also one of the main risk factors for impairment of patients’ overall quality of life after surgery [14]. A recent report by the Canadian Institute for Health Information showed that patients scheduled for day surgery in Canadian teaching hospitals return to the emergency department or are readmitted within 30 days postoperatively at incidence rates of 9.4% and 7.3%, respectively [15]. The additional costs associated with these cancellations and unplanned readmissions amount to several billion dollars every year [15].

In efforts to ensure better perioperative support and follow-up for patients undergoing day surgeries, many tools have been developed [16]. With recent technological advances and with the widespread use of smartphones and tablets, digital solutions have been suggested to guide and follow these patients [17].

The department of anesthesiology at the Centre hospitalier de l’Université de Montréal (CHUM; University of Montreal Medical Center in English), in collaboration with LeoMed, has developed a dedicated digital smart solution specifically designed to offer personalized perioperative support and follow-up via a telecare platform that can easily be installed on any smartphone. We hypothesize that the implementation of this telehealth platform at CHUM will (1) reduce the rate of unanticipated cancellations of elective surgeries through better preoperative information and communication, (2) improve quality of life 30 days after surgery due to faster and better recognition of postoperative complications, and (3) reduce the rate of emergency department or unplanned medical visits and unnecessary calls to the health information line, as well as reduce unplanned hospital readmissions, through more personalized health support. Our overall conjecture is that including the LeoMed patient-tailored telecare platform in the perioperative day-surgery pathways of patients will improve the medicoeconomic impact of care services compared with standard care. The main objective of this study is to assess the cost-effectiveness of the LeoMed platform, and our secondary objective is to perform a cost-utility analysis.

## Methods

### Design

In order to achieve the abovementioned objectives, we propose to conduct a single-center, single-blinded, 2-group randomized controlled trial. A group of patients who use the LeoMed patient-tailored telecare platform will be compared to a group of patients who have access to usual care. As mentioned earlier, the study will be conducted at CHUM.

### Population

**Figure 1:**
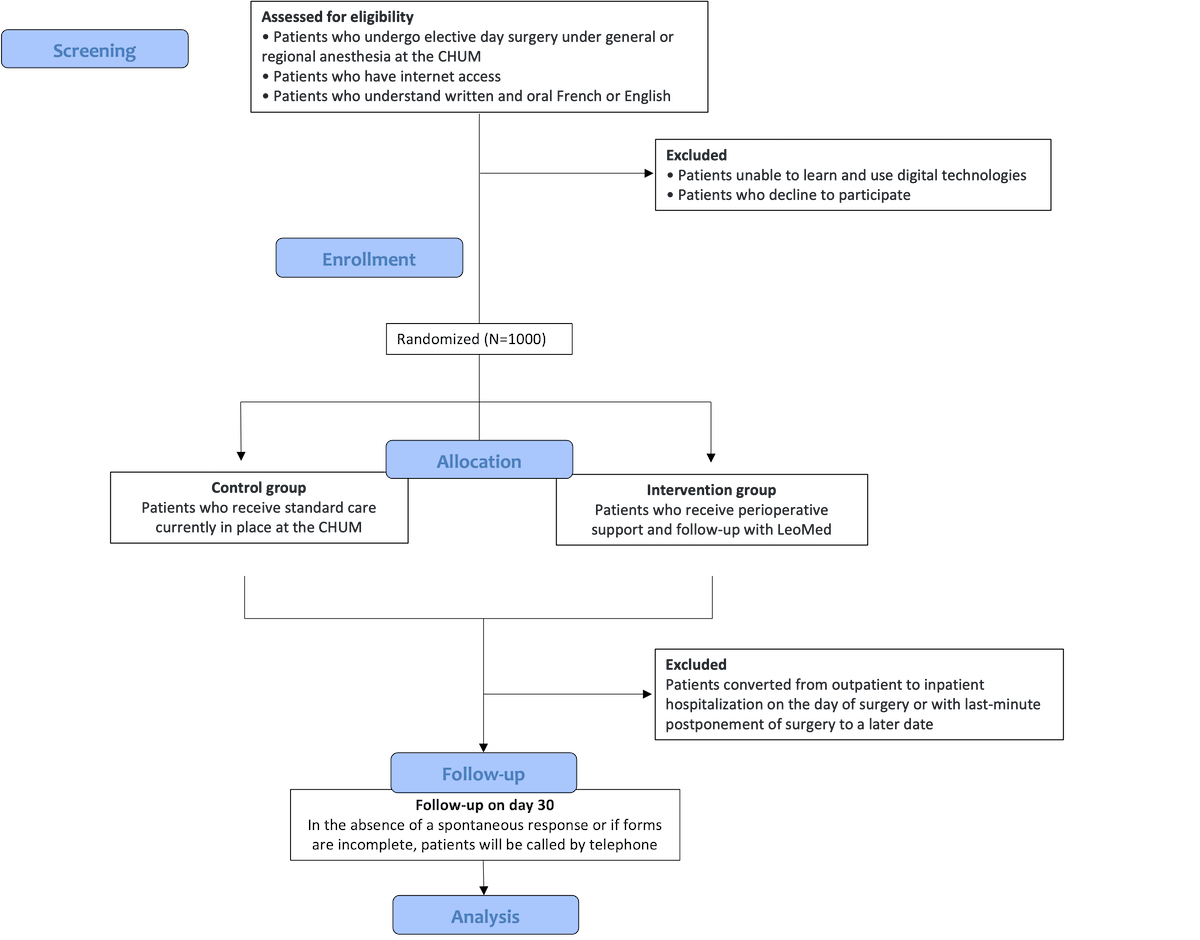
Flowchart of study participation. CHUM: Centre hospitalier de l’Université de Montréal.

All patients aged over 18 years who undergo elective day surgery under general or regional anesthesia at CHUM will be eligible. All included patients must (1) have internet access and (2) understand written and oral French or English. Patients were possibly excluded at two stages: (1) before randomization, if they were unable to learn and use the digital technologies or if they refused to participate, and (2) after randomization, in cases of conversions from outpatient to inpatient hospitalization on the day of surgery or in cases of last-minute postponement of the surgery to a later date. Figure 1 shows the trial flowchart.

### Blinding and Allocation Concealment

Eligible patients will first be approached by telephone by a member of the research team when the preoperative clinical appointment is booked. During the preoperative clinical assessment, patients will receive both oral and written information on the study by a research nurse not involved in patient care. Patients will be included in the trial only after a research nurse has obtained written informed consent.

All included patients will be electronically randomized into the intervention group or the control group. Randomization will be performed by one of the investigators using the MedSharing Randomizer for Clinical Trial web application. The 2 groups will be balanced at a 1:1 ratio. Patients who are recruited but do not receive surgery within the study time frame will be secondarily excluded.

The study will be single-blinded. Statisticians will assess the primary and secondary outcomes, blinded to the randomization group. However, due to the nature of the intervention, neither the patients nor the care providers will be blinded to the randomization.

### Recruitment and Intervention

During the recruited patients’ visits to the preoperative clinic, they will benefit from safe and restricted web access to the LeoMed telehealth platform. After confirmation of the date of surgery, patients will be allocated according to the randomization list to the intervention group or the control group.

### Intervention Group

The day before their surgery, patients will receive an email or SMS notification encouraging them to access the LeoMed platform. The platform prompts patients to click on links that direct them to the digital portal of the CHUM website, where all health sheets describing the perioperative pathway are published and grouped together, and preoperative psychological empowerment video resources are available that have been specifically created with the help of the CHUM communications service.

Postoperatively, notifications will be sent to the participants every day during the first 4 days. These notifications will direct patients to online forms assessing their medical condition. The questionnaires used are derived from the Brief Pain Inventory [18] and the Institut national de santé publique du Québec (INSPQ; Quebec Public Health Expertise and Reference Center in English) [19], which suggests using a simple questionnaire with 4 items for identification of surgical site infections. The items include redness at the surgical site, presence of pus at the surgical site, fever, and wound pain.

The LeoMed platform will automatically classify patients’ responses using an artificial intelligence algorithm and generate alerts on the home page. The home page is displayed on a giant screen on CHUM’s telehealth service, which will offer tailored support according to patients’ clinical need.

Health support will be provided by CHUM’s telehealth service, which comprises a team of 50 nurses. This department is a 24/7 call center that manages many telecare platforms dedicated to remote patient support for various medical specialties. All the staff of this service are trained in new technologies. Their mission is to provide health care remotely and to coordinate the interventions of different medical and paramedical personnel within the hospital. Specifically for this project, patient responses appear on a giant screen in the main open space where the CHUM telehealth service nurses work. In the event of an alert, a nurse will contact the patient within a maximum of 8 hours and provide appropriate care.

On postoperative day (POD) 15 and 30, the LeoMed platform will send a follow-up form that aims to evaluate all indicators considered in the medicoeconomic analysis. All the indicators are detailed in Table 1.

**Table 1:** Schedule of enrollment, interventions, and assessments.

Timepoint			Preoperative consultation		At the confirmation of the date of surgery		Preoperative day 1		POD^a^ 1		POD 4		POD 7		POD 15 and POD 30
**Enrollment**															
	Eligibility screening	✓													
	Informed consent	✓													
	Allocation			✓											
**Intervention**															
	Intervention group	✓				✓		✓		✓		✓		✓	
	Control group	✓												✓	
**Assessments on** **the LeoMed platform**															
	Quality of life (EQ-5D-5L visual analogue scale)	✓												✓	
	Unplanned readmissions													✓	
	Unplanned visits (eg, family doctor, walk-in clinic)													✓	
	Emergency department visits													✓	
	Calls to CHUM^b^ online support													✓	
	Calls to the Quebec health information line													✓	
	**Assessments from the electronic medical record system**														
	Same-day cancellations							✓							
	Calls to CHUM online support													✓	

^a^POD: postoperative day.

^b^CHUM: Centre hospitalier de l’Université de Montréal.

### Control Group

Participants assigned to the control group will be followed up according to usual care, which consists of a telephone call the day before the procedure as a reminder of safety instructions. Postoperative follow-up is not standardized and remains specific to each surgical specialty. Access to a limited version of the LeoMed platform will also be given to these participants during the preoperative clinic visit. This version includes a preoperative questionnaire and follow-up forms at POD 15 and POD 30 that are identical to those received by the intervention group and collect the indicators considered in the primary analysis (Table 1). Like the patients in the intervention group, the control patients will have 24/7 access to the provincial or CHUM health information lines, if needed.

### Primary Outcome

The primary outcome will be the cost-effectiveness of the LeoMed telecare platform deployment assessed by its direct costs, including unanticipated cancellations on the day of surgery due to preoperative instructions not being followed, calls to the local CHUM health information line, calls to the Quebec health information line (ie, 811), visits to the emergency department, and unplanned readmissions or medical visits (to family doctors or outpatient clinics) for problems related to the procedure within the first 30 days after the procedure.

### Secondary Outcome

The secondary outcome considered for the cost-utility analysis will be quality-adjusted life years (QALYs). According to a recent update of the guide for economic evaluation published by the French National Authority for Health [20], the EQ-5D-5L visual analog scale (VAS) is the preferred measure used to assess quality of life and to derive utility values for use in health technology appraisals. It will be assessed at POD 30 and compared with the preoperative baseline [21]. The EQ-5D-5L VAS is a questionnaire that is well validated. It includes 5 dimensions, each describing a different aspect of health: mobility, self-care, usual activities, pain/discomfort, and anxiety/depression. Patient satisfaction with care services will also be assessed.

### Data Collection

Data for primary and some secondary outcome measures will be collected from the LeoMed platform until POD 30, with the exception of same-day cancellations. These data will be collected on POD 1 from the electronic medical record (EMR) system by 1 member of the research team (Table 1). In the absence of spontaneous responses from patients at POD 30 or if the forms are incomplete, patients will be called by one of the research assistants to assess the indicators included in the cost-effectiveness and cost-utility analyses. If a postoperative event is reported via the form, the patient will also be contacted personally by telephone.

For each patient, data will be systematically registered and archived on a local secure server. Patients will be identified using a unique numeric code, and all patient data will be anonymized to ensure confidentiality. All data will be extracted and cross-referenced with Oacis (Telus Health), the EMR system used at CHUM.

### Data Analysis

Data analysis will be performed by a team of researchers at the École des Hautes Études Commerciales in Montreal, Quebec, using SPSS (version 28; IBM Corp). All data will be analyzed according to the intention-to-treat principle. Descriptive statistics will be reported as frequencies and proportions for categorical data and means and standard deviations for continuous variables. Demographic data for the 2 groups of patients will be compared using the Mann-Whitney *U* test (for ordinal data), the chi-square test (for nominal data), and a 2-tailed *t* test (for continuous data).

Considering the Canadian guidelines for the economic evaluation of health technologies [22], the perspective of the health care system will be privileged in this study. Specifically, 2 main types of analyses will be carried out. First, all interventions in both groups related to surgery (calls to the provincial or CHUM information lines, visits to the ambulatory unit, hospital readmissions, and visits to the emergency department) will be recorded during the study to perform the cost-effectiveness analysis. Second, a cost-utility analysis will be conducted to quality-weight each effectiveness outcome measured in QALYs. QALYs will be calculated as life with the health state under study multiplied by the utility associated with that health state. CHUM’s internal data will be used to determine the cost of health care staff and the average cost of an emergency room visit for the clientele concerned in fiscal year 2019-2020. For hospitalizations, we will use the average costs calculated according to the American diagnosis-related groups method, which was applied at CHUM in 2019-2020. Technology costs will be estimated based on license prices in effect at the time of the study and amortized on a straight-line basis over a 5-year period. All calculations will be in Canadian dollars. Last, patient satisfaction with care services will be measured in both groups at the end of the trial using Likert scales adapted from Sicotte et al [23]. This protocol follows the SPIRIT (Standard Protocol Items: Recommendation for Interventional Trials) 2013 Statement for trial protocols [24].

### Sample Size

There are very few studies that have examined the effects of digital solutions for personalized support and follow-up for patients undergoing day surgery. We based our sample size calculations on Jaensson et al [25], assuming that a better postoperative recovery (ie, decreased symptoms and complications) should reduce the number of postoperative calls to the health information line, visits to the emergency department, unplanned readmissions and medical visits, and the associated costs (our primary outcome). Jaensson et al [25] reported better recovery (based on the global Swedish Web Version of the Quality of Recovery [SwQoR] score) in an intervention group (ie, day surgery patients using a recovery assessment mobile phone app) compared with a control group (standard care) on POD 7 (effect size 0.21; *P*<.001). With a significance level of .05 and a statistical power of .90, G*Power (Heinrich Heine University) generated a group size of 357. To allow for a 20% rate of loss to follow-up and protocol deviations, we thus aim to recruit 500 patients per arm. In the third quarter of 2019, CHUM provided nearly 630 outpatient surgical stays per month on average. Considering 70% of patients should be eligible for the study [26], it will take about 12 months to recruit 1000 patients.

### Ethics Approval

The study conforms to the principles outlined in the Helsinki Declaration. The project has been approved by the institutional review board and research ethics committee at CHUM (21.110). The trial was registered at the ClinicalTrials.gov (NCT04948632). If any protocol amendments are needed, research committees and the institutional review board at CHUM will be asked to approve them. Participant recruitment began in August 2022.

## Results

The platform was launched in January 2022. A patient-as-partner approach was chosen in the initial phase, where 12 patients who had undergone day surgery in the last 6 months were recruited to test the platform. Another 12 patients scheduled for outpatient surgery tested the optimized support and follow-up platform. Their feedback helped to correct and improve the platform before beginning study recruitment. The inclusion of the first patients began in August 2022. As of October 31, 2022, we enrolled 142 patients.

## Discussion

### Principal Findings

Outpatient surgeries have been significantly increasing in the last decade in Canada, with more than 1.8 million interventions being performed annually [2]. These surgeries were historically restricted to low-risk interventions, but recent improvements in anesthesia care and the development of minimally invasive surgical techniques have resulted in moving some higher-risk interventions to day-surgery pathways. Limitations in the number of hospital beds due to the COVID-19 pandemic have probably accelerated the transition toward outpatient surgery. Nowadays, major procedures, including intra-abdominal, intrathoracic, orthopedic, and endovascular surgeries, are commonly performed as ambulatory surgeries [27-29].

In addition, the number of elderly patients undergoing same-day surgery is set to increase. In 2010, the American Ambulatory Surgery survey showed that among patients who underwent day surgery, 35% and 15% were aged 65 years or older and 75 years or older, respectively [30]. The burden of comorbidities in patients undergoing day surgery also has changed. Nowadays, patients with American Society of Anesthesiologists Physical Status III and IV are commonly eligible. These older patients with more comorbidities are sent home on the day of surgery, raising questions about quality of recovery and prognosis.

To resolve these issues, digital health solutions represent an attractive option. They have been highlighted by the World Health Organization as a potential lever to increase quality of health, enhance equality of care, and reach more patients, including those in rural areas [31]. As mentioned earlier, LeoMed’s telehealth platform is specifically designed to offer perioperative support and follow-up for outpatient care. The platform was developed by the LeoMed company in partnership with members of the anesthesia department at CHUM to meet the challenges of patients’ perioperative day-surgery pathways.

One challenge is ensuring optimal communication of the measures that should be followed preoperatively to promote rapid postoperative recovery. In our institution, patients are informed of these preoperative measures during their visit at the preoperative clinic. The average time between this preoperative visit and the surgery is often one to several weeks. In the intervention group, we again provide all the documents containing the useful preoperative information in a text message sent the day before the intervention. Ideally, we would have liked to provide this information 48 to 72 hours in advance to give patients more time to read and assimilate it. Unfortunately, the operating program is confirmed only on the day before surgery due to institutional COVID-19 policy.

In the postoperative context, several studies have shown that telehealth reduces the use of opioids and improves quality of recovery [25,32,33]. We previously showed that the prevalence of moderate to severe postoperative pain after ambulatory surgery ranges from 21% to 30% at 24 hours, and as much as 24% to 43% at 7 days [12]. Inadequate pain relief is one of the main risk factors for impairment of patients’ overall quality of life after surgery [14] and can lead to inappropriate postoperative use of opioids [13]. Yet, to our knowledge, no study has demonstrated solid economic effects at the health-system level [34,35]. Hence, the lack of medicoeconomic data does not encourage government institutions and public medical insurance to recognize and promote the use of mobile technologies for outpatient surgeries. This trial aims to fill this gap.

### Limitations

We propose to conduct a randomized controlled trial that is single-center, which represents the main limitation of our study protocol. Indeed, we will not be able to generalize our findings to ambulatory centers that perform other types of surgical procedures, specifically minor procedures. This limitation must be balanced with the difficulty of developing and implementing the same telehealth solution in many hospitals. Moreover, technical challenges, costs, and data security issues might represent barriers to the successful implementation of similar telehealth solutions in other settings.

### Conclusions

This trial will help determine the cost-effectiveness of telehealth when used in the perioperative context. The trial is also necessary because it will improve knowledge about postoperative pain and adverse effects after day surgery. Outpatient surgery will not remain at low risk of complications for long. Nowadays, many procedures previously requiring hospitalization are evolving toward day-surgery pathways, and monitoring of potential adverse events is mandatory to control associated costs. This study will provide a clearer view of patient-reported outcome measures after outpatient surgery.

## Abbreviations

CHUM: Centre hospitalier de l’Université de Montréal
EMR: electronic medical record
INSPQ: Institut national de santé publique du Québec
POD: postoperative day
QALY: quality-adjusted life years
VAS: visual analogue scale

## Data Availability

The data sets generated during and/or analyzed during this study will be made available from the corresponding author on reasonable request.

## References

[1] (2011). Trendwatch Chartbook 2011: Trends Affecting Hospitals and Health Systems. American Hospital Association.

[2] (2007). Trends in Acute Inpatient Hospitalizations and Day Surgery Visits in Canada, 1995-1996 to 2005-2006. Canadian Institute for Health Information.

[3] (2000). The NHS Plan: A Plan for Investment, A Plan for Reform. National Health Service.

[4] Walsh M (2018). Improving outcomes in ambulatory anesthesia by identifying high risk patients. Curr Opin Anaesthesiol.

[5] Leslie RJ, Beiko D, van Vlymen Janet, Siemens DR (2013). Day of surgery cancellation rates in urology: Identification of modifiable factors. Can Urol Assoc J.

[6] Kaddoum R, Fadlallah R, Hitti E, El-Jardali Fadi, El Eid G (2016). Causes of cancellations on the day of surgery at a tertiary teaching hospital. BMC Health Serv Res.

[7] Bailey CR, Ahuja M, Bartholomew K, Bew S, Forbes L, Lipp A, Montgomery J, Russon K, Potparic O, Stocker M (2019). Guidelines for day-case surgery 2019: Guidelines from the Association of Anaesthetists and the British Association of Day Surgery. Anaesthesia.

[8] Jouffroy L, Guidat A, Coustets B (2010). Prise en charge anesthesique des patients en hospitalisation ambulatoire. Ann Fr Anesth Reanim.

[9] (2021). Chapter 6: Guidelines for the Provision of Anaesthesia Services for Day Surgery 2021. Royal College of Anaesthetists.

[10] Fortier J, Chung F, Su J (1998). Unanticipated admission after ambulatory surgery--a prospective study. Can J Anaesth.

[11] Rosén Helena Inger, Bergh IH, Odén Anders, Mårtensson Lena Birgitta (2011). Patients' experiences of pain following day surgery - at 48 hours, seven days and three months. Open Nurs J.

[12] Carlier J, Robin F, Pages N, Quinart A, Roy M, Pauchard J, Quintana I, Nouette-Gaulain K (2021). Pain evaluation after day-surgery using a mobile phone application. Anaesth Crit Care Pain Med.

[13] Richebé Philippe, Brulotte Véronique, Raft Julien (2019). Pharmacological strategies in multimodal analgesia for adults scheduled for ambulatory surgery. Curr Opin Anaesthesiol.

[14] Katz N (2002). The impact of pain management on quality of life. J Pain Symptom Manage.

[15] (2013). All-cause readmission to acute care and return to the emergency department. Canadian Institute for Health Information.

[16] Dahlberg K, Jaensson M, Nilsson U (2019). "Let the patient decide" - Person-centered postoperative follow-up contacts, initiated via a phone app after day surgery: Secondary analysis of a randomized controlled trial. Int J Surg.

[17] Forget P, Dahlberg K (2021). Is multi-source feedback the future of perioperative medicine?. Anaesth Crit Care Pain Med.

[18] Poundja J, Fikretoglu D, Guay S, Brunet A (2007). Validation of the French version of the brief pain inventory in Canadian veterans suffering from traumatic stress. J Pain Symptom Manage.

[19] (2014). Surveillance des infections du site opératoire : outils et méthodologies pour les milieux de soins. Institut National de Santé Publique du Québec.

[20] (2012). Choices in methods for economic evaluation. Department of Economics and Public Health Assessment of France.

[21] Herdman M, Gudex C, Lloyd A, Janssen M, Kind P, Parkin D, Bonsel G, Badia X (2011). Development and preliminary testing of the new five-level version of EQ-5D (EQ-5D-5L). Qual Life Res.

[22] (2017). Guidelines for the economic evaluation of health technologies: Canada. Canadian Agency for Drugs and Technologies in Health.

[23] Sicotte C, Paré Guy, Morin S, Potvin J, Moreault M (2011). Effects of home telemonitoring to support improved care for chronic obstructive pulmonary diseases. Telemed J E Health.

[24] Chan A, Tetzlaff JM, Altman DG, Laupacis A, Gøtzsche Peter C, Krleža-Jerić K, Hróbjartsson Asbjørn, Mann H, Dickersin K, Berlin JA, Doré Caroline J, Parulekar WR, Summerskill WS, Groves T, Schulz KF, Sox HC, Rockhold FW, Rennie D, Moher D (2013). SPIRIT 2013 statement: defining standard protocol items for clinical trials. Ann Intern Med.

[25] Jaensson M, Dahlberg K, Eriksson M, Nilsson U (2017). Evaluation of postoperative recovery in day surgery patients using a mobile phone application: a multicentre randomized trial. Br J Anaesth.

[26] (2017). Portrait numérique des foyers québécois. Centre facilitant la recherche et l’innovation dans les organisations (CEFRIO).

[27] Abaza R, Martinez O, Ferroni MC, Bsatee A, Gerhard RS (2019). Same day discharge after robotic radical prostatectomy. J Urol.

[28] Duceppe E, Harlock J, Elkouri S, Dubois L, Parlow J, Parekh R, Tandon V, Belley-Cote EP, Spence J, Borges FK, Devereaux P (2021). Major cardiovascular events following endovascular aneurysm repair. J Am Coll Cardiol.

[29] Hoeffel DP, Daly PJ, Kelly BJ, Giveans MR (2019). Outcomes of the first 1,000 total hip and total knee arthroplasties at a same-day surgery center using a rapid-recovery protocol. J Am Acad Orthop Surg Glob Res Rev.

[30] Hall M, Schwartzman Alexander, Zhang Jin, Liu Xiang (2017). Ambulatory surgery data from hospitals and ambulatory surgery centers: United States, 2010. Natl Health Stat Report.

[31] (2019). WHO guideline: recommendations on digital interventions for health system strengthening. World Health Organization.

[32] Semple JL, Sharpe S, Murnaghan ML, Theodoropoulos J, Metcalfe KA (2015). Using a mobile app for monitoring post-operative quality of recovery of patients at home: a feasibility study. JMIR Mhealth Uhealth.

[33] Pronk Y, Peters MCWM, Sheombar A, Brinkman J (2020). Effectiveness of a mobile eHealth App in guiding patients in pain control and opiate use after total knee replacement: randomized controlled trial. JMIR Mhealth Uhealth.

[34] Dahlberg K, Philipsson A, Hagberg L, Jaensson M, Hälleberg-Nyman M, Nilsson U (2017). Cost-effectiveness of a systematic e-assessed follow-up of postoperative recovery after day surgery: a multicentre randomized trial. Br J Anaesth.

[35] Armstrong KA, Semple JL, Coyte PC (2014). Replacing ambulatory surgical follow-up visits with mobile app home monitoring: modeling cost-effective scenarios. J Med Internet Res.

